# Allele-Specific PCR for *KRAS* Mutation Detection Using Phosphoryl Guanidine Modified Primers

**DOI:** 10.3390/diagnostics10110872

**Published:** 2020-10-26

**Authors:** Alexey S. Chubarov, Igor P. Oscorbin, Maxim L. Filipenko, Alexander A. Lomzov, Dmitrii V. Pyshnyi

**Affiliations:** Institute of Chemical Biology and Fundamental Medicine, SB RAS, 8 Lavrentiev Avenue, 630090 Novosibirsk, Russia; osc.igor@gmail.com (I.P.O.); max@niboch.nsc.ru (M.L.F.); lomzov@niboch.nsc.ru (A.A.L.)

**Keywords:** mutation detection, *KRAS* mutations, allele-specific PCR, blocker PCR, modified oligonucleotides, phosphoryl guanidine oligonucleotide (PGO)

## Abstract

Establishing the Kirsten rat sarcoma *(KRAS*) mutational status is essential in terms of managing patients with various types of cancer. Allele-specific real-time polymerase chain reaction (AS-PCR) is a widely used method for somatic mutations detection. To improve the limited sensitivity and specificity, several blocking methods have been introduced in AS-PCR to block the amplification of wild-type templates. Herein, we used a novel modified oligonucleotide with internucleotide phosphates reshaped 1,3-dimethyl-2-imino-imidazolidine moieties (phosphoryl guanidine (PG) groups) as primers and blockers in the AS-PCR method. Four common *KRAS* mutations were chosen as a model to demonstrate the advantages of the PG primers and blockers utilizing a customized PCR protocol. The methods were evaluated on plasmid model systems providing a *KRAS* mutation detection limit of 20 copies of mutant DNA in a proportion as low as 0.1% of the total DNA, with excellent specificity. PG-modification can serve as the universal additional mismatch-like disturbance to increase the discrimination between wild-type and mutated DNA. Moreover, PG can serve to increase primer specificity by a synergetic effect with additional mismatch and would greatly facilitate medical research.

## 1. Introduction

Somatic mutations play a significant role in oncogenesis. They are routinely analyzed for diagnosis and treatment at clinical laboratories worldwide. The Kirsten rat sarcoma (*KRAS*) is a crucial gene in the development of a variety of cancers [1]. The mutation status of this gene is an important characteristic of many cancer types such as pancreatic, colon, lung and ovarian tumors [1,2]. The most common *KRAS*-activating mutations arise in codons 12, 13, 61, 117 and 146 [3]. According to the COSMIC database (the Catalogue Of Somatic Mutations In Cancer), a majority (approximately 90–95%) of *KRAS*-activating mutations occur in codons 12 (p.G12A, p.G12C, p.G12D, p.G12R, p.G12S, and p.G12V) or 13 (p.G13D) of exon 2, whereas less common mutations occur in exon 3 and exon 4. Among the G12 codon mutations, the most frequent ones are G12D and G12V in colon and rectal cancers [3].

Establishing the *KRAS* mutational status of tumor samples is essential in terms of managing patients with various types of cancer. Predominantly, there are three methods including nucleic acid sequencing, real-time PCR with melt-curve analysis and allele-specific (AS) real-time polymerase chain reaction (AS-PCR), that have been utilized to detect mutations in the *KRAS* gene [1,2]. At present, sequencing technologies range from classical di-deoxy-Sanger sequencing to next-generation sequencing (NGS) for mutation detection [4,5,6,7]. NGS technology provides high-throughput and base pair-resolution data, good sensitivity and permits the analysis of multiple mutations simultaneously [7,8,9]. However, NGS has a much longer analysis time and relatively high per-sample costs. Thus, it is not cost-effective for routine determination of *KRAS* in a tumor sample [7,8,9]. PCR with melt-curve analysis is a rapid turnaround, low cost and less labor-intensive method [10]. However, it can detect mutations in samples containing 1% to 10% cells with mutated DNA [1,10].

In AS-PCR, wild-type or mutated DNA molecules are selectively amplified using an AS primer, where the nucleotide substitution site is located at the 3′-end of the primer, i.e., the amplification efficiency of the mismatch allele is lower than that of the perfect match allele. The advantages of this method are low cost, high sensitivity (~1% of allele frequency is detectable) and single closed tube performance (preventing contamination). However, the use of AS-PCR in *KRAS* mutation detection is restricted by low discrimination efficiency between mutant and wild-type DNA for several types of mismatches [2,11]. A further limitations is that only targeted mutations can be determined, with lack of specificity driven by combining mismatch bases and formation of primer dimers. Therefore, laborious optimization of the AS-PCR conditions and primer structure is desirable for each new mutation [12]. To increase detection sensitivity and specificity for the mutation, various blocking oligonucleotides have been introduced in AS-PCR to decrease the efficiency of wild-type DNA amplification [2,12,13,14]. 

**Figure 1:**
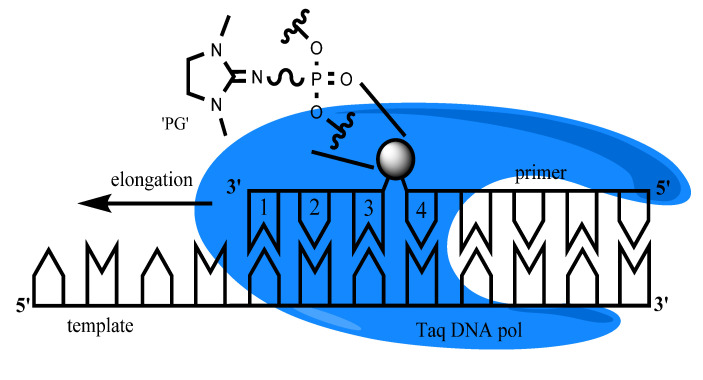
Taq-polymerase and template/primer complex schematic representation with the primer 3′-end nucleotide numeration. Sphere indicates phosphoryl guanidine (PG) modification of internucleoside phosphate moiety.

Over the previous decade, nucleic acid analogues have been extensively described to improve diagnostic analysis by PCR. Several of them, including peptide nucleic acids (PNA), locked nucleic acids (LNA), phosphorothioate, zip nucleic acids (ZNA), methyl phosphotriester oligonucleotide analogues (MPTE), nonnatural bases isoguanine (iG) and 5′-methylisocytosine (iC), are currently used for real-time PCR applications [2,15,16,17,18,19,20,21]. For instance, LNA is widely used for the detection of mutations with miRNA and DNA methylation [2]. Several of these modifications (MPTE, PNA, LNA) due to their physicochemical properties (a neutral charge, conformation, etc.) can bind to the complementary single-stranded nucleic acids. Therefore, they have higher thermal stability of duplexes [2,16,22]. Recently, a new type of uncharged phosphate-modified nucleic acid analog, namely phosphoryl guanidine oligonucleotides (PGOs), has been described [23,24]. A phosphoryl guanidine group may be easily introduced into any desired position of an oligonucleotide by standard automated DNA synthesis using phosphoramidite chemistry. In this class of compounds, replacement of the negatively charged oxygen atom with an electroneutral 1,3-dimethylimidazolidine-2-imine group (Figure 1) increases the hydrophobicity of the oligonucleotide while maintaining good water solubility, forms thermodynamically stable duplexes with DNA, but does not disturb the DNA double helix structure [25]. Previously, PGOs were shown to exhibit resistance to some nucleases [26] and can be used as promising tools for different biological applications, including acting as primers for DNA amplification in PCR [27,28,29,30,31,32,33].

In the current work we investigated the possibility of using PGO analogs as primers for AS-PCR and for the allele-specific competitive blocker PCR (ASB-PCR) to increase discrimination efficiency between mutant and wild-type DNA. Several most common *KRAS* mutations in codons 12 and 13 (p.G12A, p.G12D, p.G12V, and p.G13D) were used as models to validate the concept. The protocol described herein is standardized with AS-PCR using TaqMan probes for amplification detection. The sensitivity and selectivity of PCR assays have been evaluated on plasmid templates, harboring the aforementioned *KRAS* mutations or the wild-type *KRAS* fragment. It has been discovered that primers with one and three PG-modifications are suitable for AS-PCR and ASB-PCR, respectively. Methods are straightforward in assay optimization and can achieve 0.1% sensitivity with excellent specificity in the detection of *KRAS* mutations. The cumulative effect of the PG-modified primers and PG-blockers has shown great potential for applications in ASB-PCR.

## 2. Materials and Methods

### 2.1. Synthesis and Isolation of Oligonucleotides

Oligonucleotides were synthesized in an ASM-800 automated synthesizer (Biosset, Russia) according to the standard protocol of the 2-cyanoethyl phosphoramidite method using commercially available deoxyribonucleoside monomers and appropriate porous glass (Glen Research, Sterling, VA, USA). Oligonucleotides containing phosphoryl guanidine units were synthesized using the protocol described previously by LLC NooGen [23,24]. Oligonucleotides were purified by RP-HPLC performed in the Agilent 1200 series chromatograph (Agilent, Santa Clara, CA, USA) on a column (4.6 × 150 mm) containing the Eclipse XDB-C18 sorbent (5 μm) (Agilent, USA) with a 0–90% linear gradient of acetonitrile concentration in 0.02 M triethylammonium acetate solution for 30 min at a flow rate of 1.5 mL/min. Fractions containing the target product were evaporated in vacuo. The bulk of triethylammonium acetate was removed by coevaporations with ethanol. To remove the protecting dimethoxytrityl group, the isolated oligonucleotides were treated with 80% acetic acid (25 °C, 7 min). Purified oligonucleotides were concentrated following by precipitation with 2% LiClO_4_ in acetone, washing with pure acetone, and desiccation under vacuum. After desiccation, the oligonucleotides were dissolved in 0.1 mL of deionized water and stored at –20 °C. The concentration of oligonucleotides in stock solutions were determined in accordance with the working process described above [26].

### 2.2. Plasmid Standards

The control plasmids contained a partial sequence of the wild-type *KRAS* gene or *KRAS* gene with mutations in codons 12 and 13 (p.G12A, p.G12D, p.G12V and p.G13D) serving as positive controls, were used to assess method sensitivity and were constructed by Shanghai RealGene Bio-tech, Inc (Shanghai, China). Before use, all control plasmids were purified, linearized by digestion with BamHI restriction endonuclease and quantified using NanoDrop Lite A4 spectrophotometer (Thermo Fisher Scientific, Waltham, MA, USA).

### 2.3. Real-Time PCR

Real-time PCR assays were performed in 20 µL containing 1× PCR-buffer (65 mM Tris–HCl, pH 8.9, 24 mM (NH_4_)_2_SO_4_, 0.05 % Tween-20, 3 mM MgSO_4_), 0.2 mM dNTP, 450 nM primers, 0–2250 nM blockers, 100 nM fluorescent hydrolysis probe, DNA template (exact amount indicated below), and 1 U of Taq-polymerase (Biosan, Novosibirsk, Russia). A control plasmid was used as a DNA template at the concentration indicated below. Amplification was carried out in a CFX96 Real-Time PCR Detection System (Bio-Rad, Hercules, CA, USA) according to the following program: 95 °C for 3 min followed by 45 cycles of 95 °C for 10 s, and 60 °C for 40 s with a collection of fluorescent signals at the FAM channel. Reactions were carried out at least in triplicate and performed several times on separate occasions.

Average Cq ± standard deviation (SD) values are given in the tables. PCR analysis was performed using a reverse primer (k-rev) 5′-CATATTCGTCCACAAAATGATTCTG-3′, probe 5′-FAM-CTGTATCGTCAAGGCACTCTTGC-BHQ1-3′ and a series of forward primers 5′-AAACTTGTGGTAGTTGGAGXXXX-3′ (codon 12), 5’-GTGGTAGTTGGAGCTGXXXX-3′ (codon 13). XXXX—four nucleotides of the primer 3′-terminus, which are presented in the text as an abbreviation of the whole primer. Boldly marked nucleotides represent mismatched nucleotides in the relation to the wild-type DNA sequence. The symbol “*” in primers sequence means phosphate group modified with 1,3-dimethylimidazolidine-2-imine residue (phosphoryl guanidine (PG) modification). PG modification presented in Figure 1. 

In each PCR plate forward primer 5′-GACTGAATATAAACTTGTGGTAGTTG-3′ was used as a reference primer (k-ref) to compare the data between various PCR experiments. Corresponding ΔCq values were calculated and used for the further analysis of the primer’s efficacy. NTC—no template control.

## 3. Results and Discussion

### 3.1. Effect of the Number of PG Groups and Their Position from 3′ End of the Primer on the AS-PCR Results

Allele-specific amplification, combined with fluorescence probe real-time polymerase chain reaction (real-time AS-PCR), has been widely used for detecting genetic variants, mutations, or single nucleotide polymorphisms (SNPs). The region of the DNA template that interacts with a polymerase via the minor groove of the duplex structure is about 6-8 nucleotides long and is located at the 3′-terminus of the primer strand [34]. In principle, a mutation can be detected using AS-PCR primers based on the 3′-terminal nucleotide of a primer that corresponds to a specific mutation site. To obtain reliable discrimination between wild-type (WT) DNA and DNA with a mutation, AS primers usually have additional nucleotide substitutions within a 2–4 nucleotides region closest to the 3′-end [11,35,36]. To examine the usefulness of phosphoryl guanidine oligonucleotides (PGOs) as primers for AS-PCR, we utilized previously established sequences for *KRAS* mutation detection [35,37]. PCR assays were evaluated on plasmids harboring *KRAS* gene fragments of wild-type or with four of the most common mutations in 12-13 codons (p.G12A, p.G12D, p.G12V, and p.G13D).

The influence of the number of PG groups, and their position at 3′-terminus, were investigated using AS-primers CT**TC** which showed in preliminary experiments the best level of discrimination when used without any modifications (Table 1) and with WT and *KRAS* G12A mutation plasmids as templates. Bold symbols marked nucleotides represent mismatched nucleotides in relation to the WT DNA sequence, here T/G and C/G substitutions (WT DNA sequence CTGG).

It was discovered that four (*C*T*T*C) and three (C*T*T*C) PG groups at the 3′-end of the primer fully inhibited PCR. The latter fact is in a good correlation with previously obtained results of the authors [32,33]. The Cq value for two PG-modified primers depended on the modification position from the primer’s 3′-end and decreased in the following order: CT*T*C ≥ *CTT*C > C*T*TC > *C*T**TC** > C*TT*C (Table 1). However, for most of the AS-primers Cq was higher than 35 cycles which indicated an unacceptably low PCR efficiency and is not recommended for use in AS-PCR. The Cq value for single PG-modified primers was much lower. The distancing of the modification from the 3′-end of the primer led to a decrease in Cq value. No amplification with wild-type *KRAS* plasmid as a template was observed for all PG-modified primers, indicating an excellent specificity. The lowest Cq values with appropriate selectivity were discovered for the primers with PG modification at the third and/or fourth internucleoside phosphate (Table 1). For comparison, the same results were obtained using the primer sequence CTG**C** without additional mismatch at the second nucleotide from the 3′-end (Table S1).

### 3.2. Allele-Specific PG-Modified Primer Improves Discrimination of Mutant and WT DNA

As mentioned above, the major limitation of AS-PCR is a lack of primer specificity to discriminate between WT and mutant DNA for some types of mismatches. In the case of somatic mutation detection, the situation is significantly complicated by the heterogeneity of the sample, which may contain both normal and tumor cells leading to low-frequency mutant DNA in the abundant WT DNA background. To investigate the possibility of increasing specificity to discriminate between WT and mutant DNA, primers with PG modification at the third and/or fourth internucleotide phosphate were used as they demonstrated the best performance (lowest Cq values) in the previous experiment. As a control for AS-PCR, a reference primer (k-ref) was used and also primers that had previously been used for *KRAS* mutation detection (C**G**G**A**, G**A**G**A**, CT**AT**, CT**TC**) [35,37]. During the experiment, the portion of mutant plasmid DNA was reduced to obtain decreased ratios of mutant to WT DNA, based on a total DNA amount of 2 × 10^4^ copies per reaction (Table 2). Primers with PG modification at fourth phosphate from 3′-end usually have significantly higher Cq values (cf. C*T**AT** and *CT**AT**, Table 2) which are more than 40 cycles. This could be caused by impaired interaction of Taq-polymerase thumb amino acids (Arg536 and Ser515) with PG-modified phosphate residue in the fourth position [34]. This may lead to the significantly lower coordination of the modified nucleic acid complex by protein. Such high values of Cq do not apply to AS-PCR. For the primers with PG modification at the third internucleotide phosphate for each mutation detection, ΔCq_(WT−1%)_ values (ΔCq = Cq(WT) − Cq(1%) for the same primer, Cq(WT)—cycle quantification value obtained using WT DNA, Cq(1%)—cycle quantification value obtained using 1% mutant DNA on the background of WT DNA) were much higher than the native primers, which indicates better discrimination efficiency. However, in the case of the primer G*AG**A** the Cq value was three cycles higher than for the primer without the PG group (Table 2). Due to this case, the primer’s sequence design is desirable.

### 3.3. Simultaneous Efficiency of PG Modification and Mismatches in the Primers Structure

Choosing additional mismatches to increase primer specificity has been a challenge for AS-PCR, and criteria for designing primers are still unclear. To identify the influence of the simultaneous effect of the mismatch and PG modification, mismatched nucleotides at second and/or third position from the 3′-end of the primers were taken randomly (Table 3). It had been described before [34] that an additional mismatch at the third position from the 3′-end proved to be the most effective in increasing the discrimination efficiency without significant loss of PCR efficiency (unpublished data). Nevertheless, the second nucleotide position can be effective in a particular nucleotide context [36]. To evaluate sensitivity and selectivity, DNA from mutant plasmids was diluted into WT plasmids (total DNA amount of 2 × 10^4^ copies per reaction). The proportion of mutant plasmid DNA was gradually reduced to obtain decreasing ratios of mutant to WT DNA.

For G12A mutation detection, an increase occurred in discriminating ability with the introduction of an additional mismatch: 1.5, 5.7, 4.7 of ΔCq_(WT−1%)_ for CTG**C**, CT**TC**, C**A**G**C** primers, respectively. For G12V, G12D and G13D mutations detection, the same results were registered. This indicates the mutation detection additional mismatch within the 2–3 nucleotides closest to 3′-end is preferable. However, the second mismatch gave the improvement only in several cases (Table 3). Remarkably, that for the primer with PG modification C*TG**C**, the ΔCq_(WT−1%)_ value was 5.8 versus 1.5 for the CTG**C** for G12A detection. In this case, the PG group worked better for specific mutant DNA detection than primers with two mismatched nucleotides, such as CT**TC** or C**A**G**C**. However, in terms of the primers G*TG**A** (G13D), C*TG**T** (G12V) and C*TG**A** (G12D), there was no significant effect of the PG modification. A possible explanation is that the obtained results principally depended on the 3′-end mismatch. Polymerases reflect decreasing discrimination of 3′-end nucleotide mismatches in the following order: Pur/Pur > Pyr/Pyr > Pur/Pyr = Pyr/Pur [11,34]. However, there have been exceptions to this general rule reported [34], which could be explained by different thermodynamic destabilization effects that strongly influence primer elongation efficiency [36]. For instance, [34] Taq DNA-polymerase doesn’t efficiently elongate a primer with a Pyr/Pyr like С/С mismatch at the 3′-end of the primer (cf. Ct values of CTG**C** and CTG**A**, CTG**T**, GTG**A** primers, Table 3). 

The simultaneous effect of the PG group and mismatches leads to the extraordinary discrimination effect for C*T**TC,** and only little benefit for the C*AG**C**. ΔCq_(WT−1%)_ values for C*T**TC** and C*AG**C** were 12.3 and 6.2, respectively (cf. ΔCq for the control primer CT**TC** was 5.7). Better results for the C*T**TC** can be explained by the SNP type. It is known [36] that for SNP type G/C (G12A mutation) the mismatches in the second base pair closest to 3′-end of primer shows the highest discrimination efficiency, followed by third. For the SNP type G/A (G12D, G13D), the best location of the mismatch was in the third site and showed the highest discrimination efficiency [36], which was confirmed by the data in Table 3. Obtained results of the WT and the mutant allele discriminating effect for G12A, G12D and G13D mutations were in a good correlation of additional mismatch and PG modification effects in the appropriate site. 

For the SNP type G/T (G12V mutation) the mismatches in the third site should have the highest discrimination efficiency followed by the second and fourth sites. However, in the case of G12V mutation, additional mismatch or PG modification in the third internucleotide phosphate didn’t lead to any significant effect of the mismatch discrimination capability. These results are likely to be related to the strong destabilization strength of mismatch types at the 3′-end of primer for G12D, G13D and G12A, while for the G12V mutation the effect was weak [36]. Therefore, primers generated from weak mismatch type C/T with mutated DNA (G12V mutation) had the lowest discrimination efficiency (Table 3). Of all the *KRAS* mutations, G12V is considered to be one of the most frequent *KRAS* codon 12 mutations in colorectal cancer patients [3]. Moreover, G12V has been related to a more aggressive phenotype, a worse progression of the disease and a short time of survival [38]. Increasing the specificity of its detection is very important for improving clinical outcome. For the *KRAS* G12V mutation detection primer CT**AT**, ΔCq_(WT−1%)_ values were only 1.5,which indicates great difficulty to achieve the appropriate results. By the simultaneous effect of the two unpaired nucleotides and PG modification (C*T**TT,** C*T**AT**), much higher ΔCq_(WT−1%)_ values (3.9 and 5.5, respectively) were obtained. However, in comparison with other ΔCq_(WT−1%)_ values for the best PG-primers for other mutations obtained for G12V, the data is not so impressive, which can be explained by the weak destabilization strength of the 3′-end mismatch [36]. 

In qPCR, amplification efficiency is a very important parameter which can be calculated from a simple linear regression of the Cq values plotted against the log of the initial copy number. As a consequence, it is recommended that the following descriptors of the standard curve are reported for qPCR amplification: amplification efficiency (E), the linear regression coefficient (R^2^) and especially the y-intercept value, which uniquely describes the standard curve and indicates the sensitivity of the reaction. Standard curves show the PCR efficiency values for primers for G12A mutation detection generated from the dilution series of mutant DNA, e.g., for the C*T**TC** primer E = 98.3% and R^2^ = 0.999, y-intercept = −3.3641 (Figure S1). The same results were obtained for G12V, G12D and G13D mutations detection (Figures S2 and S3).

This discovery, while preliminary, suggests that PG-modification can be used as a universal additional mismatch-like disturbance of a primer/DNA complex with good discrimination properties of DNA polymerase action. Primers used in the present experiments contained only one PG-modified nucleotide at their 3′-end. Moreover, the PG group may have served to increase primer specificity by the synergetic effect with additional mismatch and would greatly facilitate and increase the reliability of clinical diagnostics. The PG-modified primers may be synthesized using standard equipment and could be potentially obtained from commercial oligonucleotide suppliers in a low-cost way.

### 3.4. Detection Limit and Specificity Investigation of the Synthesized PG-Modified Primers

**Figure 2:**
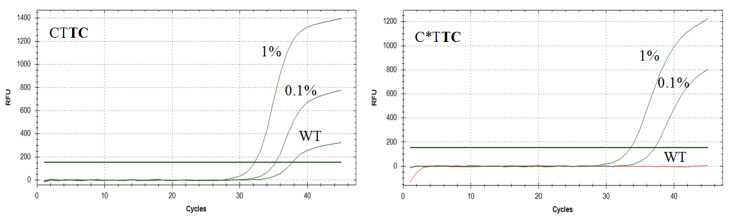
Typical amplification curves obtained in allele-specific PCR assays. *KRAS* G12A mutation detection was performed using CT**TC** and C*T**TC** primers and WT DNA (2 × 10^4^ copies per reaction), 1% and 0.1% of mutant DNA in the background of WT DNA.

Using plasmid DNA, mutation detection was achieved as low as 1% for each mutation. The best results in discriminating the wild-type and the mutant allele and low Cq values were found for primers C*T**AT,** C*T**TT** (G12V mutation), С*T**TC** (G12A), C*GG**A** (G12D)**,** G*AG**A,** G*CG**A** (G13D) (Table 3). Typical amplification curves obtained from allele-specific PCR of the native and PG modified primers are presented in Figure 2.

To determine assay reproducibility, several experiments were performed in five replicates with various amounts of the mutant template in the samples (SD value~±0.5, Table S2). The next section of the survey was concerned with extended research of the primers with reliable WT discrimination efficiency. The primers were tested to achieve lower than 1% detection sensitivity with good specificity in the abundant wild-type DNA background. A standard real-time PCR protocol and PG modified primers were used to obtain ΔCq_(WT−0.1%)_ values (Table 4). The best result were observed for both *KRAS* G12A and G13D followed by G12D mutation assays, which showed 0.1% detection sensitivity (~20 copies per reaction) without nonspecific amplification on WT DNA. However, the absolute Cq values for all modified primers were ≥35 cycles. It should be noted that for the primers with and without PG modification сlose Cq values were obtained in most cases. These data suggest that 0.1% of mutant DNA detection can be achieved through assay optimization.

### 3.5. Improving AS-PCR Specificity by Wild-Type DNA Blocking PCR

**Figure 3:**
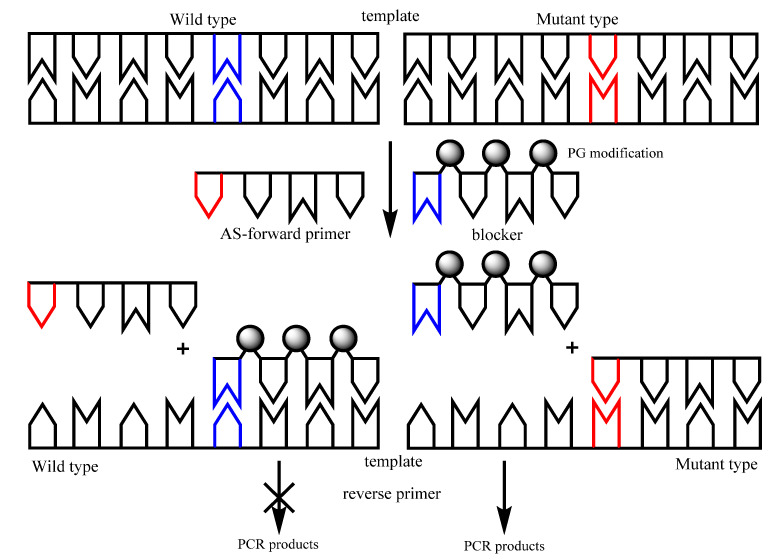
A schematic representation of wild-type blocking PCR. Wild-type variant is marked by blue, mutant—by red. AS-forward, reverse primer and one WT-blocking primer constitute the PCR reaction mixture. On the 3′-terminus of the blocking primer, inhibiting three PG groups have been attached to prevent extension by DNA polymerization. The AS-primer overlapped with the blocking primer at the target mutation site and was in competition with the blocking primer.

Previous studies [32,33] have shown an ability of PGO to terminate enzymatic DNA synthesis. In the present work, a simple approach was described using PG-modified primers to facilitate the mutations detection by blocking WT DNA amplification. The concept of this technique is similar to classical wild-type blocking PCR assays (ASB-PCR) (Figure 3). This approach was tested using several *KRAS* mutations. As AS-primers, the best in discriminating the WT and the mutant allele primers were chosen (Table 4). The blocking primers had the sequence C*T*G*G for 12 codon and G*T*G*G for 13 codon, which are very similar to AS-primers but lack any nucleotide substitutions at the 3′-end. Therefore, blocker oligonucleotides are perfectly matched to the WT DNA. 

**Figure 4:**
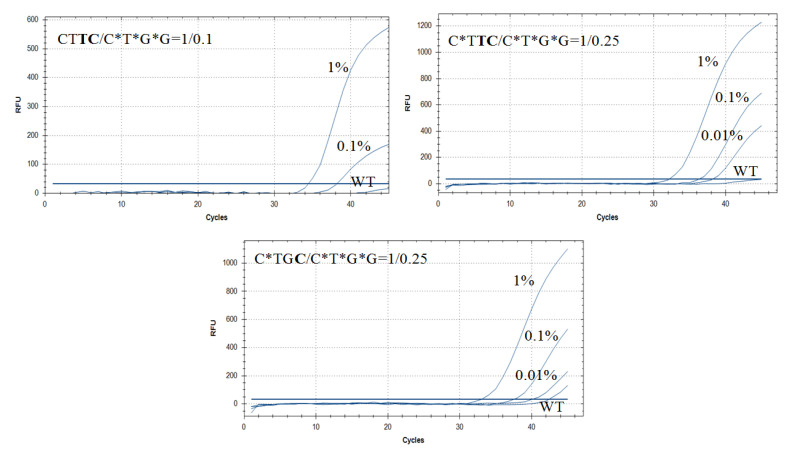
Typical amplification curves obtained in allele-specific blocking PCR assays. *KRAS* G12A mutation detection was performed using CT**TC**, C*T**TC** and C*TG**C** primers and C*T*G*G blocker and WT DNA (2 × 10^4^ copies per reaction), 1%, 0.1% and 0.01% mutant DNA in the background of WT DNA.

The competition-based ASB-PCR approach inherently suffers from lower PCR efficiency with the stronger WT DNA suppression. To obtain good discrimination between wild-type and mutated DNA for a certain type of blocker, very high 10 [39] or 20-fold [40] excess over AS-primers is required. For other systems such as T-blockers [41] or PNA [39], five and 1.2 blocker excesses are sufficient. In the current work, several combinations of AS-primers and blockers were tested to illustrate the largest Cq separation between the mutant and WT reactions. In the reaction mixture, the concentration of AS-primer was constant. The blocking primer performance was sensitive to the amount of blocking primer added. More than equimolar excess of the PG-blocker to an AS-primer inhibited PCR (Table S3). Therefore, fixed primer to blocker ratios 1:0.5, 1:0.25, and 1:0.1 were studied (Table S3). As the AS-primer:blocker ratio increased, the difference between 1% mutant and WT DNA became more pronounced and could clearly be distinguished when the ratio reached 1:0.25 as seen in Table 5 and Table S3. The ratio 1:0.5 partly inhibited PCR for most primers. Keeping blocker concentration at the minimum required amount is a good rule. The best results were obtained using primer/blocker ratios 1:0.25 and/or 1:0.1 (Table 5 and Table S3). In most cases, the system had higher discrimination efficiency and similar Cq values as the reaction without a blocker. For instance, for the primer CT**TC**, Cq_(1%)_ values were 31.5 and 33.5 (with blocker). ΔCq_(WT−1%)_ values were 5.2, and much higher with the blocker (11.5) which shows increasing mutation selectivity detection. Using 2 × 10^4^ copies of DNA per reaction, no amplification was observed. The (Cq(WT) value was N/A, which made the calculation of ΔCq_(WT−1%)_ impossible. Taking a higher number of recombinant DNA molecules (2 × 10^5^ copies) the Cq value could be calculated (Table 5). Typical amplification curves obtained from allele-specific blocking PCR assays are presented in Figure 4.

Using primers with PG modification, it is possible to reach better discrimination ability of a PCR. For С*T**TC** (G12A mutation) and C*T**TC/**C*T*G*G, ΔCq_(WT−1%)_ values were 12.4 and up to 14.2, respectively. At the same time, Cq(1%) values for CT**TC** and C*T**TC/**C*T*G*G were almost the same: 31.5 and 32.5, respectively. The same results were obtained for the G**A**G**A** primer/blocker system. For the worst discriminated C12V mutation, the primer/blocker assay gave an excellent result in discriminating the WT and the mutant allele. ΔCq_(WT−1%)_ values for CT**AT,** C*T**AT**, C*T**AT**/C*T*G*G were 1.0, 5.5 and 9.0, respectively. These data present clear supporting evidence that PG-blockers play a strong WT DNA suppression role. The cumulative effect of the PG-modified primers and PG-blockers has great potential for applications in ASB-PCR.

Blocking methods have been applied successfully in several studies [2,12,13,14,41] to increase detection sensitivity and specificity for mutation detection. High sensitivity has been achieved in the assay, reaching one copy of mutant plasmid DNA to 1000 wild-type DNA dilution, corresponding to a fractional abundance of 0.1% (Table 5). ΔCq_(WT−0.1%)_ values for the primers/blockers system are ranked in the 5–9 cycles range, which indicates good WT suppression. Recent studies [13,41] have shown sensitivity improvement down to 0.1% of mutant DNA by adopting various primer/blocker ratios, too. Compared to the assays presented in other works [13,41], PG-blockers have some advantages. It is not necessary to design the blocker structure since it can be used by the full complementary to WT DNA primer with three 3′-terminal PG-modifications. Cq values of 0.1% detection have been 37–39 [41] and 36–37 [13] which are comparable to the values in the present work. 

Several studies have detected 0.01% of mutant DNA for the detection of *KRAS* using a highly optimized WT blocking PCR approach [42,43] or a combination of PCR and direct sequencing methods [44]. Comparing the results for the PG-primers and PG-primers/PG-blockers, it can be observed from Table 4 and Table 5 that it is possible to obtain 0.1%, and a bit lower detection sensitivity, with excellent discrimination between mutant/WT DNA by ASB-PCR assay. However, we failed to reach 0.01% sensitivity with sufficient mutant/WT DNA discrimination due to the amount of DNA being close to the theoretical limit of qPCR detection. The differences of the Cq_WT_ and Cq_0.01%_ show the potential to obtain 0.01% sensitivity in the presence of more DNA copies per reaction, which represents a remarkably artificial case during analyzing natural samples. As colorectal cancer (CRC) is the most studied with regard to *KRAS* mutation, due to the implementation of anti-EGFR (epidermal growth factor receptor) antibodies into clinical practice, it has been considered that around 2% of patients with metastatic CRC truly harbor *KRAS* mutations with a low mutant allele frequency. While the percent of *KRAS*-positive tumors increases with the increase of the assay sensitivity, it can be explained by artifacts from sample fixation, e.g., formalin treatment for FFPE (formalin-fixed, paraffin-embedded) specimens. Therefore, the high sensitivity of the assay (<1%) itself has its pitfalls and may lead to false-positive results, and such samples with a low mutant allele frequency should be a subject of a more scrupulous analysis with additional procedures, namely, UDG (uracil-DNA glycosylase) treatment [7]. Nevertheless, by adapting the primer/blocker ratio, it is possible to improve the assay in each case to yield optimal Cq and ΔCq values. Using a higher DNA amount, as in previous studies [42,43], it is possible to obtain over 0.1% sensitivity.

## 4. Conclusions

PG-modification is a noncharged organic residue at the internucleotide phosphodiester fragment which does not significantly alter the secondary structure of oligonucleotide complexes with DNA [26]. However, the presence of the large organic residue (18 N,C,H-atoms instead of a single oxygen atom) in the tight DNA/enzyme interaction site can significantly influence the efficiency and selectivity of the enzymatic reaction. Increased selectivity of DNA-polymerases has been demonstrated during the use of primers modified at the internucleoside phosphodiester residue [16,17,34]. In the current work, PG-modified primers for AS-PCR and an allele-specific competitive blocker PCR (ASB-PCR) for *KRAS* mutations (G12V, G12A, G12D and G13D) detection, was presented.

Our results suggested that the PG-modified primers and blockers are advanced tools for AS-PCR or ASB-PCR methods. The incorporation of PG modification into PCR primers leads to the identification of single mismatches by AS-PCR and ASB-PCR assays with a good specificity without significant losses in the sensitivity in the abundant wild-type DNA background. Moreover, PG can serve as the universal additional mismatch-like disturbance to increase primer specificity and would greatly facilitate medical research. The method is straightforward for assay optimization and has been evaluated on plasmid model systems, providing a *KRAS* mutation detection limit of 20 copies of mutant DNA in a proportion as low as 0.1% of the total DNA, without nonspecific WT DNA amplification. A limitation of the proposed *KRAS* assay is that PG-modification, in some cases, leads to an increase in the Cq value, and partly inhibits PCR that leads to a decrease in PCR efficiency. In the future, it is hoped to use the properties of PGOs to establish optimal reproducible PCR technology in each laboratory, which may stimulate further research and assist clinicians.

## Figures and Tables

**Table 1:** Allele-specific real-time polymerase chain reaction (AS-PCR) *KRAS* G12A mutation detection using mutant DNA mixed with wild type (WT) DNA (total 2 × 10^5^ copies per reaction) at various ratios (% of mutant DNA).

Primers	Cq		
	WT	1%	100%
CT**TC**	34.25 ± 0.05	28.7 ± 0.1	21.72 ± 0.02
C*T**TC**	N/A	29.65 ± 0.07	23.8 ± 0.2
CT*TC	N/A	33.24 ± 0.01	26.8 ± 0.2
CTT*C	N/A	33.5 ± 0.1	26.67 ± 0.01
C*TT*C	N/A	36.4 ± 0.2	29.2 ± 0.1
*C*T**TC**	N/A	37.9 ± 0.1	32.0 ± 0.1
C*T*TC	N/A	40.9 ± 0.2	34.8 ± 0.1
*CTT*C	N/A	N/A	40.1 ± 0.3
CT*T*C	N/A	N/A	N/A

No template control (NTC) was undetermined in all the reactions, N/A indicates that no Cq was obtained for a typical 45-cycle reaction. Symbol “*” means phosphoryl guanidine (PG) modification location. Boldly marked nucleotides mean mismatched nucleotides in relation to the WT DNA sequence.

**Table 2:** AS-PCR *KRAS* mutation detection using WT DNA (total 2 × 10^4^ copies per reaction) and 1% mutant DNA in the background of WT DNA.

Primers		Cq	
		WT	1%
G12V	CT**AT**	33.4 ± 0.1	31.9 ± 0.1
	C*T**AT**	38.7 ± 0.3	33.2 ± 0.1
	*CT**AT**	N/A	40.2 ± 1.3
G12D	C**G**G**A**	35.8 ± 0.1	32.50 ± 0.05
	C*GG**A**	41.7 ± 1.8	32.9 ± 0.1
	*C**G**G**A**	N/A	42.9 ± 1.2
G12A	CT**TC**	37.7 ± 0.3	32.0 ± 0.2
	С*T**TC**	N/A	32.7 ± 0.2
G13D	G**A**G**A**	36.3 ± 0.1	32.3 ± 0.1
	G*AG**A**	N/A	35.0 ± 0.1
	*G**A**G**A**	N/A	39.2 ± 0.3

No template control (NTC) was undetermined in all the reactions, N/A indicates that no Cq was obtained for a typical 45-cycle reaction. Symbol “*” means PG modification location. Boldly marked nucleotides represent mismatched nucleotides in relation to the WT DNA sequence.

**Table 3:** AS-PCR *KRAS* mutation detection using WT DNA (total 2 × 10^4^ copies per reaction) and 1% mutant DNA in the background of WT DNA.

Primers		Cq		ΔCq	Primers		Cq		ΔCq
		WT	1%	Cq_WT_ − Cq_1%_			WT	1%	Cq_WT_ − Cq_1%_
G12A	CTG**C**	33.0 ± 0.5	31.5 ± 0.1	1.5	G12V	CTG**T**	26.2 ± 0.1	26.0 ± 0.1	0.2
	С*TG**C**	38.4 ± 0.4	32.6 ± 0.1	5.8		C*TG**T**	30.7 ± 0.1	29.7 ± 0.1	1.0
	CT**TC**	37.7 ± 0.3	32.0 ± 0.2	5.7		CT**AT**	33.4 ± 0.1	31.9 ± 0.1	1.5
	С*T**TC**	N/A	32.7 ± 0.2	12.3 ^a^		C*T**AT**	38.7 ± 0.3	33.2 ± 0.1	5.5
	C**A**G**C**	37.7 ± 0.2	33.0 ± 0.1	4.7		CT**TT**	35.3 ± 0.1	34.2 ± 0.1	1.1
	C*AG**C**	38.7 ± 0.3	32.5 ± 0.1	6.2		C*T**TT**	41.8 ± 1.1	37.9 ± 0.3	3.9
G13D	GTG**A**	29.0 ± 0.1	28.1 ± 0.1	0.9		C**G**G**T**	28.0 ± 0.1	27.4 ± 0.1	0.6
	G*TG**A**	33.4 ± 0.1	32.3 ± 0.1	1.1		C*GG**T**	28.5 ± 0.1	27.6 ± 0.1	0.9
	G**A**G**A**	36.3 ± 0.2	32.3 ± 0.1	4.0		C**C**G**T**	31.7 ± 0.1	30.0 ± 0.1	1.7
	G*AG**A**	N/A	35.0 ± 0.1	10.0 ^a^		C*CG**T**	33.9 ± 0.2	30.9 ± 0.1	3.0
	G**C**G**A**	35.0 ± 0.1	31.0 ± 0.1	4.0	G12D	CTG**A**	27.7 ± 0.1	27.0 ± 0.1	0.7
	G*CG**A**	N/A	33.1 ± 0.1	11.9 ^a^		C*TG**A**	29.2 ± 0.1	28.7 ± 0.1	0.5
	G**G**G**A**	38.0 ± 0.4	32.8 ± 0.1	5.2		C**G**G**A**	35.8 ± 0.1	32.50 ± 0.05	3.3
	G*GG**A**	N/A	36.4 ± 0.2	8.6 ^a^		C*GG**A**	41.7 ± 1.8	32.9 ± 0.1	8.8

No template control (NTC) was undetermined in all the reactions, N/A indicates that no Cq was obtained for a typical 45-cycle reaction. Symbol “*” means PG modification location. Boldly marked nucleotides represent mismatched nucleotides in relation to the WT DNA sequence. SNP G/C (G12A), G/T (G12V), G/A (G12D, G13D); 3′-end mismatch site with mutated DNA C/C, Pyr/Pyr (G12A); C/T, Pyr/Pyr (G12V); C/A, Pyr/Pur (G12D, G13D). ^a^ If Cq(WT) = N/A to calculate ΔCq = Cq(WT) − Cq(1%) value Cq(WT) = 45 has been used.

**Table 4:** *KRAS* mutations detection by AS-PCR using various mutant/wild-type DNA ratios (total 2 × 10^4^ copies per reaction) and the several best-obtained primers for each mutation detection.

Primers		Cq			ΔCq	
		WT	0.1%	1%	Cq_WT_ − Cq_0.1%_	Cq_WT_ − Cq_1%_
G12A	СT**TC**	37.7 ± 0.3	35.1 ± 0.1	32.0 ± 0.2	2.6	5.7
	С*T**TC**	N/A	36.9 ± 0.2	32.7 ± 0.2	8.1 ^a^	12.3 ^a^
G12V	CT**AT**	33.4 ± 0.1	33.2 ± 0.1	31.9 ± 0.1	0.2	1.5
	C*T**AT**	38.7 ± 0.4	37.1 ± 0.2	33.2 ± 0.1	1.6	5.5
G12D	C**G**G**A**	35.8 ± 0.1	35.1 ± 0.1	32.5 ± 0.1	0.7	3.3
	C*GG**A**	41.7 ± 1.8	36.2 ± 0.2	32.9 ± 0.1	5.5	9.2
G13D	G**A**G**A**	36.3 ± 0.1	34.6 ± 0.1	32.3 ± 0.1	1.7	4.0
	G*AG**A**	N/A	38.5 ± 0.3	35.0 ± 0.1	6.5 ^a^	10.0 ^a^
	G**C**G**A**	35.0 ± 0.1	34.3 ± 0.1	31.0 ± 0.1	0.7	4.0
	G*CG**A**	N/A	37.0 ± 0.3	33.1 ± 0.1	8.0 ^a^	11.9 ^a^

No template control (NTC) was undetermined in all the reactions, N/A indicates that no Cq was obtained for a typical 45-cycle reaction. Symbol “*” means PG modification location. Boldly marked nucleotides represent mismatched nucleotides in relation to the WT DNA sequence. ^a^ If Cq(WT) = N/A to calculate ΔCq = Cq(WT) − Cq(1% or 0.1%) value Cq(WT) = 45 was used.

**Table 5:** ASB-PCR *KRAS* mutations detection of various mutant/wild-type DNA ratios (total 2 × 10^4^ or 2 × 10^5^ copies per reaction).

Primers		Cq				ΔCq	
						Cq_WT_ − Cq_1%_	
		2 × 10^4^ Copies				2 × 10^4^	2 × 10^5^
		1%	0.1%	0.01%	WT	Copies	
G12A	No blocker CTGG	24.6 ± 0.2	24.8 ± 0.2	25.0 ± 0.3	25.0 ± 0.2	0.4	-
	Blocker C*T*G*G	N/A	N/A	N/A	N/A	-	-
	No blocker CT**TC**	31.5 ± 0.1	33.9 ± 0.1	34.2 ± 0.2	36.7 ± 0.2	5.2	5.7
	CT**TC/**C*T*G*G = 1/0.1	33.5 ± 0.1	38.0 ± 0.3	N/A	N/A	11.5 ^a^	10.7
	C*T**TC/**C*T*G*G = 1/0.25	32.5 ± 0.1	36.3 ± 0.3	38.2 ± 0.4	N/A	12.5 ^a^	14.2
	C*TG**C/**C*T*G*G = 1/0.25	33.1 ± 0.1	37.5 ± 0.2	40.1 ± 0.5	42.4 ± 1.5	9.3	9.2
G12V	No blocker CT**AT**	30.6 ± 0.1	30.8 ± 0.2	31.4 ± 0.2	31.6 ± 0.2	1.0	-
	C*T**AT /**C*T*G*G = 1/0.1	36.0 ± 0.2	37.5 ± 0.4	40.3 ± 1.0	N/A	9.0 ^a^	8.8
G12D	No blocker C**G**G**A**	33.2 ± 0.1	36.0 ± 0.3	37.0 ± 0.5	37.1	3.8	-
	C*GG**A/**C*T*G*G = 1/0.1	33.6 ± 0.1	37.1 ± 0.6	39.2 ± 0.9	N/A	11.4 ^a^	9.8
G13D	No blocker GTGG	23.5 ± 0.1	24.0 ± 0.2	24.1 ± 0.1	24.2 ± 0.3	0.7	-
	Blocker G*T*G*G	N/A	N/A	N/A	N/A	-	-
	No blocker G**A**G**A**	31.1 ± 0.1	33.1 ± 0.2	33.6 ± 0.3	35.2 ± 0.2	4.1	-
	G**A**G**A/**G*T*G*G = 1/0.1	32.7 ± 0.1	34.8 ± 0.2	N/A	N/A	12.3 ^a^	8.8
	G*AG**A/**G*T*G*G = 1/0.1	35.3 ± 0.4	39.2 ± 0.8	41.0 ± 1.3	N/A	9.7 ^a^	10.9

No template control (NTC) was undetermined in all the reactions, N/A indicates that no Cq was obtained for a typical 45-cycle reaction. Symbol “*” means PG modification location. Boldly marked nucleotides represent mismatched nucleotides in relation to the WT DNA sequence. Real-time PCR assay was done using a constant 450 nM AS-primers concentration and several blocker primers ratios. ^a^ If Cq(WT) = N/A to calculate ΔCq = Cq(WT) − Cq(1%) value Cq(WT) = 45 was used.

## Supplementary Materials

The following are available online at https://www.mdpi.com/2075-4418/10/11/872/s1, Figure S1**:** Cq values of *KRAS* G12A mutation detection using C*TG**C**, *CTG**C**, *C*TG**C**, CT**TC**, C*T**TC**, k-ref primers and various amount of mutant DNA per reaction. Standard curves showing the PCR efficiency values for the several primers generated from a dilution series of mutant DNA; Figure S2**:** Cq values of *KRAS* G12V, G12D mutations detection using CT**AT**, C*T**AT**, *CT**AT**, C**G**G**A**, C***G**G**A**, *C**G**G**A** primers and various amount of mutant DNA per reaction. Standard curves showing the PCR efficiency values for the several primers generated from a dilution series of mutant DNA; Figure S3**:** Cq values of *KRAS* G13D mutations detection using G**A**G**A**, G***A**G**A**, *G**A**G**A** primers and various amount of mutant DNA per reaction. Standard curves showing the PCR efficiency values for the several primers generated from a dilution series of mutant DNA; Table S1**:** AS-PCR *KRAS* G12A mutation detection using CTG**C** and CT**TC** primers with PG-modification in the different phosphates from the 3′-end and mutant DNA mixed with wild-type DNA (total 2 × 10^5^ copies per reaction) at various ratios (% of mutant DNA); Table S2**:** AS-PCR reproducibility G12A *KRAS* mutation detection using wild-type DNA (total 2 × 10^4^ copies per reaction) and 1% mutant DNA in the background of wild-type DNA; Table S3**:** ASB-PCR *KRAS* mutations detection of various mutant/wild-type DNA ratio (total 2 × 10^4^ or 2 × 10^5^ copies per reaction).
