# Supplementary material for: Allele-Specific PCR for KRAS Mutation Detection Using Phosphoryl Guanidine Modified Primers
Source: Diagnostics (Basel). 2020 Oct 26;10(11):872. doi: 10.3390/diagnostics10110872 (PMC7692470; doi:10.3390/diagnostics10110872)
Supplement: Supplementary file 1 [file diagnostics-10-00872-s001.pdf]

Supplementary Materials

Allele-Specific PCR for KRAS Mutation Detection Using Phosphoryl Guanidine Modified Primers

Alexey S. Chubarov*, Igor P. Oscorbin, Maxim L. Filipenko, Alexander A. Lomzov and Dmitrii V. Pyshnyi*

Institute of Chemical Biology and Fundamental Medicine, SB RAS, 8 Lavrentiev Avenue, 630090, Novosibirsk, Russia; osc.igor@gmail.com (I.O.); max@niboch.nsc.ru (M.F.), lomzov@niboch.nsc.ru (A.L.)

* Correspondence: pyshnyi@niboch.nsc.ru, Tel.: +7(383)3635151 (D.P.); chubarov@niboch.nsc.ru, chubarovalesha@mail.ru; Tel.: +79137631420 (A.C.)

The influence of the number of PG groups and their position at 3′-terminus were investigated using AS-primers CT**TC** and CTG**C** (Table S1) and WT and *KRAS* G12A mutation plasmids as templates [1,2]. Bold symbols marked nucleotides means mismatched nucleotides in the relation to the wild-type DNA sequence. To determine assay reproducibility, several experiments were performed in 5 replicates with various amounts of the mutant template in the samples (Table S2). Standard curve data were generated for several native and phosphoryl guanidine (PG) modified primers for KRAS mutation detection and the PCR efficiency for each of these primers was calculated using Thermo Fisher Scientific online calculator (Figure S1-S3) [3]. All experiments had amplification efficiency more than 90% and a correlation coefficient R^2 above 0.99. Allele-specific blocking PCR KRAS mutations detection assay results using blockers containing C*T*G*G or G*T*G*G 3′-terminal fragment are presented in Table S3.

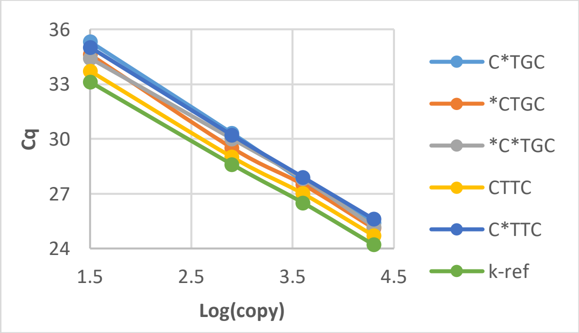

	Y-intercept	R2	qPCR Efficiency,%
C*TG**C**	-3.5603	0.9997	90.9
*CTG**C**	-3.3805	0.9981	97.6
*C*TG**C**	-3.2619	0.9986	102.7
CT**TC**	-3.2047	0.9992	105.1
C*T**TC**	-3.3641	0.9999	98.3
k-ref	-3.172	0.9998	106.7

Figure S1. Cq values of *KRAS* G12A mutation detection using C*TG**C**, *CTG**C**, *C*TG**C**, CT**TC**, C*T**TC**, k-ref primers and various amount of mutant DNA per reaction. Standard curves showing the PCR efficiency values for the several primers generated from a dilution series of mutant DNA.

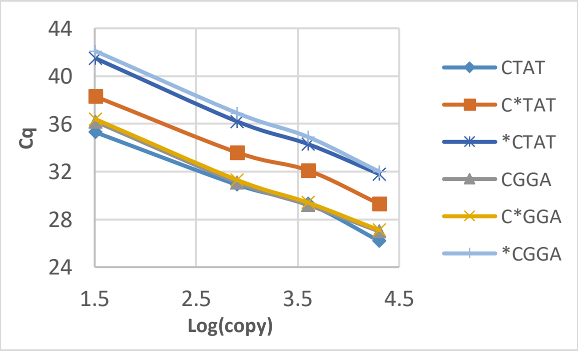

	Y-intercept	R2	qPCR Efficiency,%
CT**AT**	-3.1598	0.9901	107.2
C*T**AT**	-3.1434	0.9932	108.0
*CT**AT**	-3.4418	0.9967	95.2
C**G**G**A**	-3.2824	0.9963	101.7
C***G**G**A**	-3.311	0.9969	100.5
*C**G**G**A**	-3.5603	0.9974	90.9

Figure S2. Cq values of *KRAS* G12V, G12D mutations detection using CT**AT**, C*T**AT**, *CT**AT**, C**G**G**A**, C***G**G**A**, *C**G**G**A** primers and various amount of mutant DNA per reaction. Standard curves showing the PCR efficiency values for the several primers generated from a dilution series of mutant DNA.

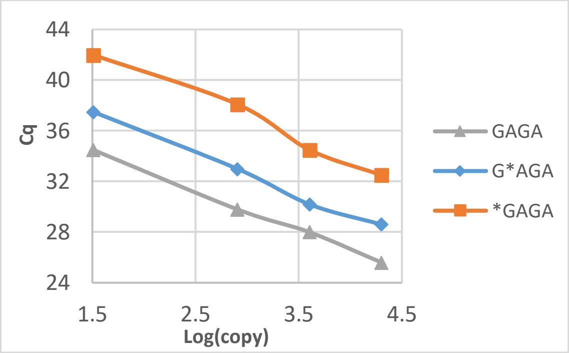

	Y-intercept	R2	qPCR Efficiency,%
**GAGA**	-3.1516	0.9979	107.6
G***AGA**	-3.2579	0.9944	102.7
***GAGA**	-3.4786	0.9852	93.9

Figure S3. Cq values of *KRAS* G13D mutations detection using **GAGA**, G***AGA**, ***GAGA** primers and various amount of mutant DNA per reaction. Standard curves showing the PCR efficiency values for the several primers generated from a dilution series of mutant DNA.

Table S1. AS-PCR *KRAS* G12A mutation detection using CTG**C** and CT**TC** primers with PG-modification in the different phosphates from the 3′-end and mutant DNA mixed with wild-type DNA (total 2×10^5 copies per reaction) at various ratios (% of mutant DNA)

**Primers**	**Cq**		
	**WT**	**1%**	**100%**
CTG**C**	28.1±0.1	27.2±0.1	24.0±0.1
C*TG**C**	33.8±0.2	28.3±0.1	21.9±0.1
*CTG**C**	37.4±0.2	28.1±0.05	22.2±0.2
*C*TG**C**	N/A	29.8±0.2	23.4±0.1
CTG***C**	36.9±0.2	30.3±0.2	24.1±0.1
CT*G**C**	36.8±0.4	31.4±0.1	25.3±0.1
*CTG***C**	N/A	37.2±0.2	27.7±0.2
C*TG***C**	N/A	34.9±0.5	29.5±0.2
CT**TC**	34.25±0.05	28.7±0.1	21.72±0.02
C*T**TC**	N/A	29.65±0.07	23.8±0.2
CT***TC**	N/A	33.24±0.01	26.8±0.2
CT**T***C**	N/A	33.5±0.1	26.67±0.01
C*T**T***C**	N/A	36.4±0.2	29.2±0.1
*C*T**TC**	N/A	37.9±0.1	32.0±0.1
C*T***TC**	N/A	40.9±0.2	34.8±0.1
*CT**T***C**	N/A	N/A	40.1±0.3
CT***T***C**	N/A	N/A	N/A

NTC (No Template Control) was undetermined in all the reactions, N/A indicates that no Cq was obtained for a typical 45-cycle reaction. Symbol "*" means PG modification location. Boldly marked nucleotides mean mismatched nucleotides in relation to the wild-type DNA sequence

Table S2. AS-PCR reproducibility G12A *KRAS* mutation detection using wild-type DNA (total 2×10^4 copies per reaction) and 1% mutant DNA in the background of wild-type DNA

**Primers**		**Cq**	
		**WT**	**1%**
k-ref	1	25.73	25.21
	2	25.53	25.23
	3	25.72	25.17
	4	25.66	24.86
	5	25.20	24.76
	aver	25.57	25.05
	SD	0.22	0.22
CT**TC**	1	36.55	31.43
	2	35.79	31.64
	3	36.17	31.66
	4	35.42	31.22
	5	36.26	31.29
	aver	36.04	31.45
	SD	0.44	0.20
C*T**TC**	1	N/A	33.13
	2	N/A	33.11
	3	N/A	32.70
	4	N/A	32.67
	5	N/A	33.00
	aver	-	32.92
	SD	-	0.22

Table S3. ASB-PCR *KRAS* mutations detection of various mutant/wild-type DNA ratio (total 2×10^4 or 2×10^5 copies per reaction)

	Primers	Cq				ΔCq Cq(WT) - Cq(1%)	
		2×10^4 copies				2×10^4 copies	2×10^5 copies
		1%	0.1%	0.01%	WT		
G12A	No blocker CTGG	24.6±0.2	24.8±0.2	25.0±0.3	25.0±0.2	0.4	-
	Blocker C*T*G*G	N/A	N/A	N/A	N/A	-	-
	No blocker CT**TC**	31.5±0.1	33.9±0.1	34.2±0.2	36.7±0.2	5.2	5.7
	CT**TC**/C*T*G*G = 1/5, 1/2	N/A	N/A	N/A	N/A	-	-
	CT**TC**/C*T*G*G = 1/1	N/A	N/A	N/A	N/A	-	-
	CT**TC**/C*T*G*G = 1/0.5	41.6±0.3	N/A	N/A	N/A	-	-
	CT**TC**/C*T*G*G = 1/0.25	37.0±0.6	40.1±1.1	N/A	N/A	-	-
	CT**TC**/C*T*G*G = 1/0.1	34.5±0.1	38.0±0.2	N/A	N/A	10.5[a]	10.7
	C*T**TC**/C*T*G*G = 1/5, 1/2	N/A	N/A	N/A	N/A	-	-
	C*T**TC**/C*T*G*G = 1/1	41.7±1.5	N/A	N/A	N/A	-	-
	C*T**TC**/C*T*G*G = 1/0.5	33.0±0.2	37.3±0.5	41.8±1.0	N/A	12.0[a]	10.1
	C*T**TC**/C*T*G*G = 1/0.25	32.5±0.1	36.3±0.3	38.2±0.4	N/A	12.5[a]	14.2
	C*T**TC**/C*T*G*G = 1/0.1	31.7±0.1	34.9±0.1	38.0±0.3	39.8±0.9	8.1	-
	C*TG**C**/C*T*G*G = 1/1	37.4±0.5	N/A	N/A	N/A	-	-
	C*TG**C**/C*T*G*G = 1/0.5	34.8±0.1	39.3±0.4	43.0±1.8	N/A	10.2[a]	9.7
	C*TG**C**/C*T*G*G = 1/0.25	33.1±0.1	37.5±0.2	40.1±0.5	42.4±1.5	9.3	9.2
	C*TG**C**/C*T*G*G = 1/0.1	32.3±0.1	36.4±0.3	38.7±0.7	39.0±1.0	6.7	-
G12V	No blocker CT**AT**	30.6±0.1	30.8±0.2	31.4±0.2	31.6±0.2	1.0	-
	No blocker C*T**AT**	31.5±0.1	35.3±0.2	36.7±0.4	37.3±0.5	5.8	-
	C*T**AT** /C*T*G*G = 1/0.5	41.3±1.0	N/A	N/A	N/A	-	-
	C*T**AT** /C*T*G*G = 1/0.25	39.3±0.8	N/A	N/A	N/A	-	8.8
	C*T**AT** /C*T*G*G = 1/0.1	36.0±0.2	37.5±0.4	40.3±1.0	N/A	9.0[a]	8.8
G12D	No blocker C**GGA**	33.2±0.1	36.0±0.3	37.0±0.5	37.1	3.8	-
	C***GGA**/C*T*G*G = 1/0.5	35.5±0.2	39.8±1.0	N/A	N/A	10.5[a]	-
	C***GGA**/C*T*G*G = 1/0.25	33.7±0.1	37.3±0.5	N/A	N/A	11.3[a]	9.8
	C***GGA**/C*T*G*G = 1/0.1	33.6±0.1	37.1±0.6	39.2±0.9	N/A	11.4[a]	9.8
G13D	No blocker GTGG	23.5±0.1	24.0±0.2	24.1±0.1	24.2±0.3	0.7	-
	Blocker G*T*G*G	N/A	N/A	N/A	N/A	-	-
	No blocker G**AGA**	31.1±0.1	33.1±0.2	33.6±0.3	35.2±0.2	4.1	-
	G**AGA**/G*T*G*G = 1/0.1	31.7±0.1	33.8±0.2	N/A	N/A	13.3[a]	8.8
	G***AGA**/ G*T*G*G = 1/0.5	38.8±0.7	N/A	N/A	N/A	-	9.3
	G***AGA**/ G*T*G*G = 1/0.25	36.2±0.2	43.0±1.5	N/A	N/A	8.8[a]	9.5
	G***AGA**/G*T*G*G = 1/0.1	35.3±0.4	39.2±0.8	41.0±1.3	N/A	9.7[a]	10.9

NTC (No Template Control) was undetermined in all the reactions, N/A indicates that no Cq was obtained for a typical 45-cycle reaction. Symbol "*" means PG modification location. Boldly marked nucleotides mean mismatched nucleotides in relation to the wild-type DNA sequence. Real-time PCR assay was done using a constant 450 nM AS-primers concentration and several blocker primers excess. [a]If Cq(WT) = N/A to calculate ΔCq = Cq(WT) - Cq(1%) value Cq(WT) = 45 was used.

References

1. Takeda, S.; Ichii, S.; Nakamura, Y. Detection of Kras mutation in sputum by mutant allelespecific amplification (MASA). *Hum. Mutat.* **1993**, *2*, 112–117, doi:10.1002/humu.1380020209.
2. Lang, A.H.; Drexel, H.; Geller-Rhomberg, S.; Stark, N.; Winder, T.; Geiger, K.; Muendlein, A. Optimized allele-specific real-time PCR assays for the detection of common mutations in KRAS and BRAF. *J. Mol. Diagnostics* **2011**, *13*, 23–28, doi:10.1016/j.jmoldx.2010.11.007.
3. Thermo Fisher Scientific qPCR Efficiency Calculator Available online: https://www.thermofisher.com/ru/ru/home/brands/thermo-scientific/molecular-biology/molecular-biology-learning-center/molecular-biology-resource-library/thermo-scientific-web-tools/qpcr-efficiency-calculator.html.
